# Emerging Goatpox Virus Threat in Wild Ruminants: First Documented Outbreak in the United Arab Emirates, 2024

**DOI:** 10.3390/vetsci13050480

**Published:** 2026-05-16

**Authors:** Christiana Hebel, Ajith Kumar, Sunitha Joseph, Joerg Kinne, Nissy Annie Georgy Patteril, Florian Pfaff, Bernd Hoffmann, Rolf Schuster, Francois Le Grange, Ulrich Wernery

**Affiliations:** 1 Central Veterinary Research Laboratory, Dubai 27748, United Arab Emirates; sjoseph@cvrl.ae (S.J.); drschusterrolf@outlook.com (R.S.); cvrl@cvrl.ae (U.W.); 2 His Highness Purchase and Supply Office, Dubai 27748, United Arab Emirates; 3 Institute of Diagnostic Virology, Friedrich-Loeffler-Institut, Suedufer 10, 17493 Greifswald–Insel Riems, Germany; florian.pfaff@fli.de (F.P.); bernd.hoffmann@fli.de (B.H.); 4 Wadi Al Safa Wildlife Centre, Dubai P.O. Box 27875, United Arab Emirates; lf.legrange@gmail.com

**Keywords:** goatpox virus, *Capripoxvirus goatpox*, wild ruminants, spillover infection, United Arab Emirates, sequencing, phylogenetics, wildlife disease

## Abstract

The goatpox virus (GPV) is a highly contagious pathogen primarily affecting domestic small ruminants in endemic regions of Northern Africa, the Middle East, and Asia. This study reports the first confirmed outbreak of GPV in captive wild ruminants in the United Arab Emirates (UAE). The outbreak occurred in a fenced 900-hectare mountainous reserve following a period of heavy rainfall, and Barbary sheep (*Ammotragus lervia*), Nubian ibex (*Capra nubiana*), Arabian oryx (*Oryx leucoryx*), and Scimitar oryx (*Oryx dammah*) were affected. Clinical signs included generalized cutaneous nodules, mucopurulent nasal discharge, respiratory distress, weakness, and emaciation. Over a three-month period, 71 animals died or were euthanized. Histopathological findings were consistent with GPV infection in goats, although typical inclusion bodies were missing. Real-time PCR confirmed GPV DNA in multiple tissues with a high viral genome load. Virus isolation was successful only in lamb testis cells. Whole-genome sequencing demonstrated that the isolates were genetically identical and clustered within the Central and Western Asia lineage, showing closest similarity to a Turkish field strain. The finding highlights the potential for cross-species transmission of GPV into wildlife and emphasizes the importance of surveillance, as well as molecular diagnostic and preventative vaccination strategies at the wildlife–livestock interface.

## Simple Summary

Goatpox is a serious viral disease that usually affects domestic goats and can cause high illness and death rates. It is common in parts of Africa, the Middle East, and Asia, where it leads to economic losses for farmers. Until now, infections in wild ruminants have been rarely documented. This study describes the first confirmed outbreak of goatpox in captive wild ruminants. The outbreak occurred in a fenced mountain reserve after a period of unusually heavy rain. Over three months, 71 animals died or were euthanized, including Barbary sheep, Nubian ibex, Arabian oryx, and Scimitar oryx. The affected animals showed skin nodules, nasal discharge, heavy breathing, weakness, and weight loss. Laboratory testing confirmed goatpox virus infection, and genetic analysis showed the virus was closely related to strains in Central and Western Asia. The source of the infection could not be determined, but indirect contact with domestic livestock or insect transmission could have played a role. This outbreak highlights the risk of disease transmission between livestock and wildlife and emphasizes the importance of surveillance and preventative vaccination in surrounding domestic animals.

## 1. Introduction

The goatpox virus (GPV; Species: *Capripoxvirus goatpox*), a highly contagious and often devastating viral disease primarily affecting domestic goats, poses a significant and ongoing challenge to livestock health and agricultural economies worldwide [1]. It is a notifiable disease according to the World Organization for Animal Health (WOAH) [2]. Goatpox disease is characterized by generalized skin lesions, fever, decreased milk production, and significant mortality rates and is a major concern to farmers. This is especially true in regions where it is endemic, such as Northern Africa, the Middle East, and Asia, where it exerts a substantial economic impact through direct losses from morbidity and mortality, as well as indirect losses from reduced productivity and trade restrictions [3].

GPV belongs to the *Capripoxvirus* genus, a group within the Poxviridae family, which includes lumpy skin disease virus (LSDV) in cattle and sheep pox virus (SPV) [4]. A critical aspect complicating disease control efforts is the cross-species infectivity observed in some strains of SPV and GPV, which are capable of infecting both sheep and goats [5].

Susceptible animals typically contract GPV through direct contact, with an incubation period of 8–13 days, though clinical signs can emerge as early as 4 days post-exposure. Mechanical transmission by insects may also play a significant role in spreading the virus, particularly in warm and humid environments. Common clinical signs of GPV infections include the development of skin lesions like papules, firm swellings, and characteristic pox lesions, and secondary infections like pasteurellosis, accompanied by high fever and respiratory distress with mucopurulent rhinitis. While morbidity rates can be very high (75–100%), mortality rates are often higher at a later stage of the disease and can reach up to 50% in fully susceptible flocks and 100% in young animals, depending on the virulence of the specific viral strain as well as the host’s immune status [5].

Traditionally associated with domestic livestock, there is growing concern about GPV’s potential to spread to wild ruminant populations. Such spillover events could lead to significant ecological consequences, impacting wildlife conservation efforts and potentially creating new reservoirs for the virus.

This article explores the clinical manifestations, transmission patterns, and economic impact of GPV, as well as the growing concern of GPV as a persistent threat to both domestic and wild caprine populations. It emphasizes the urgent need for enhanced surveillance and reliable diagnostic tools, and well-coordinated control strategies. Additionally, it describes a notable outbreak of GPV reported for the first time in the United Arab Emirates (UAE), following a period of heavy rainfall.

## 2. Materials and Methods

### 2.1. Study Site and Animals

An outbreak of GPV was investigated in a fenced mountainous reserve of approximately 900 hectares in the UAE. The reserve housed 1100 Barbary sheep (*Ammotragus lervia*), 215 Nubian ibex (*Capra nubiana*), 35 Arabian oryx (*Oryx leucoryx*), 62 Scimitar oryx (*Oryx dammah*), 23 Black buck (*Antilope cervicapra*), 44 Dorcas gazelles (*Gazella dorcas*), 6 Rhim gazelles (*Gazella leptoceros*), and 5 Arabian sand gazelles (*Gazella marica*).

### 2.2. Clinical Investigation and Case Definition

The animals were monitored for the occurrence of cutaneous lesions, respiratory signs, and general conditions. Two Barbary sheep and one Arabian oryx presenting with severe lethargy, extensive skin lesions, respiratory distress, emaciation, and poor prognosis were euthanized for animal welfare reasons and further investigations, as representatives of the affected animals. The carcasses were then transported in a cooled van to the Central Veterinary Research Laboratory (CVRL) in Dubai and subjected to detailed post-mortem and laboratory examinations.

### 2.3. Necropsy

Necropsies were performed on the two euthanized Barbary sheep (designated as cases 33 and 34) and one Arabian oryx (designated as case 4), at CVRL in Dubai. Representative samples of skin, lung, kidney, and lymph nodes were collected for further in-house examinations.

### 2.4. Histopathology

Tissue samples from all major organs were fixed in 10% neutral buffered formalin and processed for routine histopathological examination. After fixation, tissues were dehydrated through graded alcohols, embedded in paraffin wax, and sectioned at 4 µm thickness. Sections were subsequently stained with hematoxylin and eosin (H&E) and examined microscopically.

### 2.5. Virus Isolation

Tissue samples (skin papules, lung lesions, and lymph nodes) were collected from two Barbary sheep and one Arabian oryx for virus isolation on cell cultures of lamb testis, Madin–Darby bovine kidney (MDBK) cells, Vero cells, and the chorioallantoic membranes (CAMs) of embryonated chicken eggs. No additional primary cell cultures, such as sheep kidney or sheep skin cells, were evaluated for virus isolation. Primary lamb testis (LT) cells were prepared from testicular tissue obtained aseptically from a 1–5 month-old lamb at a local slaughterhouse, following previously described methods [6]. Tissue fragments were enzymatically dissociated with 0.25% trypsin and cultured in minimum essential medium (MEM, Gibco, Grand Island, NY, USA) supplemented with 10% bovine serum and antibiotics, containing 1% penicillin–streptomycin (Sigma Aldrich, Darmstadt, Germany) and 0.3% Amphotericin B (Sigma Aldrich, Darmstadt, Germany) at 37 °C in a humified 5% CO_2_ atmosphere. LT cells at passage 3 were used for virus isolation.

After three freeze–thaw cycles, the homogenized suspension was centrifuged for 5 min at 2500 rpm. The supernatant was filtered with a 0.45 μm filter (Sartorius, Goettingen, Germany), 1.0 mL of which was inoculated into lamb testis, MDBK cells, and Vero cells grown in T75 (75 cm^2^) flasks. The inoculated cultures were incubated at 37 °C for 7–14 days and monitored for cytopathic effect (CPE). If no CPE was observed after multiple passages, the supernatant was inoculated onto fresh cell cultures. In addition, 0.1 mL of the supernatant was inoculated onto CAMs of embryonated chicken eggs (9–11 days of age) and incubated at 37 °C. After 5 days, CAMs were collected carefully and examined for the presence of characteristic pock lesions of the *Poxviridae* family on the allantois membrane.

### 2.6. DNA Extraction and Real-Time PCR

To identify and confirm the aetiological agent, original tissue material (skin, lung, and Ln. prescapularis) from one Barbary sheep and one Arabian oryx was analyzed using different real-time PCR systems for the detection of capripoxvirus DNA [7]. Briefly, 100 µL of homogenized tissue material in cell culture medium was subjected to nucleic acid extraction using the NucleoMagVET kit (Macherey-Nagel, Düren, Germany) on a semi-automated KingFisher platform (KingFisher Flex, Thermo Fisher Scientific, Waltham, MA, USA). Nucleic acids were eluted in 100 µL of elution buffer.

Extracted DNA was analyzed using three independent duplex real-time PCR assays. The real-time PCR assays used have been thoroughly validated and have been published [7]. Briefly, the pan-Capripox assay (pan-Capripox-p32-FAM (Capri-p32-Mix1-Taq-FAM) detects all capripoxviruses and was combined with an internal process control targeting β-actin (β -Actin-DNA-Mix2-HEX). The second duplex assay detects and differentiates between virulent lumpy skin disease virus (LSDV) field strains (LSDV-Field-FAM, LSDfield-ORF126-Mix11-Taq-FAM) and LSDV vaccine strains (LSDV-VAC-HEX, LSDvac-Mix5-Taq-HEX). The third duplex assay enables the detection and differentiation of sheep pox virus (SPPV; SPPV-ORF041-Mix1-MGB-FAM) and goat pox virus (GTPV; GTPV-ORF095-Mix1-MGB-HEX). As little is known about the prevalence of the various capripoxviruses in wild animals, and in order to rule out dual infections, the samples from wild animals were not only tested for sheep and goat poxviruses, but the presence of LSDV was also ruled out.

### 2.7. Whole-Genome Sequencing and Phylogenetic Analysis

Viral DNA extracted from infected lamb testis cells was submitted to Eurofins Genomics (Eurofins Genomics, Cologne, Germany) for whole-genome sequencing using Illumina technology. Viral DNA was extracted from 200 µL of cell culture supernatant as described above (PCR section). Sequencing libraries were analyzed on a NovaSeq platform using paired-end 150 bp reads (NovaSeq PE150).

The raw sequencing reads were filtered and trimmed using TrimGalore (v0.6.10) and Cutadapt (v5.2) to remove adapter contamination and low-quality sequences. The resulting trimmed reads were then assembled de novo using Megahit (v1.2.9). The assembled contigs were mapped to the GPV reference sequence MN072625 using Minimap2 (2.24) to reconstruct the complete viral genome. Open reading frames (ORFs) were predicted using Prodigal (v2.6.3), and the resulting ORFs were then compared against the non-redundant protein reference database (nr) using BLASTp (v2.17.0+). Genome annotation followed the gene nomenclature of the GPV reference sequence KC951854. The fully annotated viral genome sequences were deposited in GenBank under the accession numbers PX852058 and PX852059.

For the phylogenetic analysis, the two GPV genomes obtained from Barbary sheep and the Arabian oryx were aligned with 15 full-length GPV genomes and one full-length SPV genome retrieved from GenBank, using MAFFT (v7.490). The redundant right terminal repeat was removed from the alignment, and the region corresponding to the left terminal repeat was trimmed to match the start of the RefSeq strain “Pellor” (NC_004003.1).

A phylogenetic tree was constructed using IQ-TREE (v3.0.1) running with optimal model selection (K3Pu + F + I) and applying 10,000 ultra-fast bootstrap replicates. The phylogenetic tree was rooted at the SPV reference. Phylogenetic clusters were defined based on phylogenetic topology and node support, not by applying fixed genetic-distance cutoffs. Each cluster represents a well-supported monophyletic lineage separated from the others by internal branches.

## 3. Results

### 3.1. Outbreak Description and Species Affected

The outbreak commenced approximately six weeks after a period of heavy rainfall. At the end of May 2024, two Barbary sheep (cases 33 and 34) and one Arabian oryx (case 4) were presented to the veterinarian in charge, with generalized cutaneous nodular lesions, apathy, and nasal discharge. Within the following three months, 71 animals died or were euthanized due to GPV (Table 1). In addition to the 71 animals that died or were euthanized, a substantial number of animals exhibited clinical signs and recovered, particularly Barbary sheep. Blackbuck, Dorcas, and Rhim gazelles and Arabian sand gazelles were not affected.

### 3.2. Clinical Findings

The affected animals presented with papular to nodular cutaneous lesions, distributed across the entire body (Table 1). Respiratory distress, characterized by mucoid nasal discharge, was also observed. Animals that developed respiratory signs either succumbed or were euthanized due to poor body condition, severe weakness, and emaciation. Animals presented with only skin lesions usually recovered within 8–14 days, primarily young Barbary sheep.

### 3.3. Pathological and Histopathological Findings

**Figure 1:**
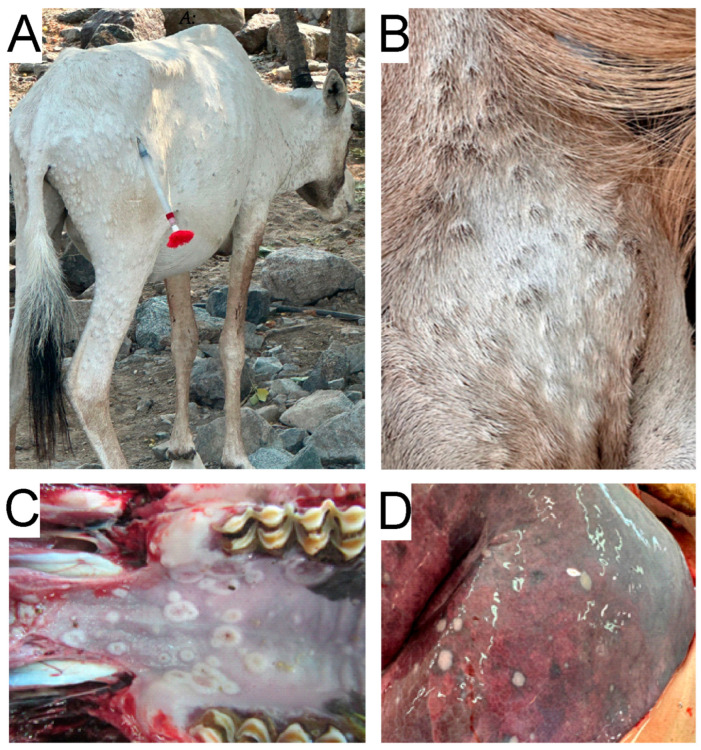
Gross lesions caused by the goatpox virus. (**A**) Arabian oryx (*Oryx leucoryx*): multiple, firm, raised skin nodules. The animal was darted in the hind leg muscles with an immobilization dart for anaesthesia, to approach the animal safely and euthanize it humanely. (**B**) Barbary sheep (*Ammotragus lervia*): multiple, firm, raised skin nodules. (**C**) Erosions and ulcers on the oral mucosa. (**D**) The displayed grey or brownish nodular subpleural lesions.

The two Barbary sheep and the Arabian oryx were presented with multiple, firm, raised skin nodules measuring 5–10 mm (Figure 1A,B). In four animals, 33 nodules were distributed across the entire body, while 34 animals had fewer nodules concentrated at the caudal end of their bodies. Common findings in all animals included erosions and ulcers on the oral mucosa and inner nostrils, measuring 5 to 8 mm, along with mucoid nasal discharge (Figure 1C). All lymph nodes were enlarged, firm, and partially hemorrhagic, and the liver was enlarged. The lungs of both sheep displayed grey or brownish nodular subpleural lesions up to 1–2 cm in diameter (Figure 1D). Both lobes were congested with alveolar emphysema and edema. No other significant lesions were observed. Histopathological investigation of the skin of 33 animals also revealed focal proliferative dermatitis with intraepidermal vesicles and a few pox-like viral inclusions. In the lungs of 3 animals, as well as 33 larger areas, exhibited suppurative to fibrinous pneumonia with bacterial presence and scattered foamy cells. For 34 animals, the lung examination revealed focal fibrinous pleuritis with underlying subpleural pulmonary necrosis, as well as eosinophilic material and foamy cells.

### 3.4. Virus Isolation

Cytopathic effects (CPE), characterized by cell rounding, were observed exclusively in lamb testis cells at passage 2. The virus isolation procedure was repeated three times with consistent results. Negative control cultures showed no cytopathic effects throughout the incubation period. Infected lamb testis cells exhibited rounding and morphological changes indicative of viral infection. However, no CPE was observed in MDBK, Vero cells, or on CAMs, even after repeated incubation passages. These findings indicate that the virus isolated from the Barbary sheep and Arabian oryx exhibited limited growth in commonly used cell lines and chorioallantois membranes, while replication was supported in lamb testis cells. Subsequent molecular analyses were therefore required for definitive virus identification and further characterization.

### 3.5. Molecular Identification by Real-Time PCR

Real-time PCR confirmed the presence of GPV DNA in all tested tissues. The corresponding Ct values obtained for the three tissue samples are summarized in Table 2. The high to very high viral loads detected in several tissues strongly support GPV infection as the cause of the severe clinical signs observed in the affected animals.

### 3.6. Genome Characterization and Phylogenetic Analysis

**Figure 2:**
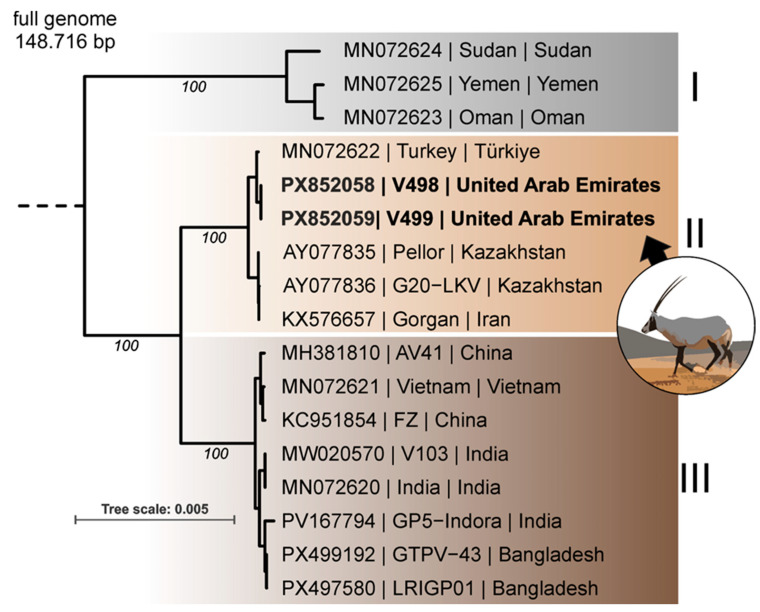
**Phylogenetic analysis of goatpox virus (GPV) genomes.** All publicly available GPV full-length genome sequences were aligned to the GPV genomes obtained from Barbary sheep and the Arabian oryx from the outbreak in wild ruminants in the United Arab Emirates. The sheeppox virus reference NC_004002 was used as the outgroup. The GPV references grouped within three phylogenetic clusters that roughly followed their geographic origin: (**I**) Northeast Africa and the southern Arabian Peninsula, (**II**) Central and Western Asia, and (**III**) South and East Asia. The sequences from the United Arab Emirates, indicated in bold, clustered with the Central and Western Asia group II. Bootstrap support is indicated for major branches in italics, and the tree scale shows substitutions per site.

Whole-genome sequencing revealed that the GPV isolates obtained from Barbary sheep and Arabian Oryx were identical. The phylogenetic analysis separated the GPV sequences into three clusters that roughly followed their geographic origin (Figure 2). The two UAE GPV isolates clustered within the Central and Western Asian lineage together with sequences from Turkey, Iran, and Kazakhstan. The genetically closest sequence, MN072622, was a GPV field isolate from Turkey and differed from the UAE isolate by only 60 bp. These differences corresponded to 50 polymorphic sites, as several repeat-associated indels involved more than one nucleotide. Comparative analysis showed that the variation consisted of 22 tandem-repeat indels and 28 SNPs, with most indels occurring in A/T-rich repeat regions. Although indels in the EGF-like growth factor, EEV membrane phosphoglycoprotein, and DNA ligase CDSs were predicted to cause frameshifts, these changes affected only the terminal regions of the proteins and are therefore unlikely to result in functional changes. Among the SNPs, transitions were more frequent than transversions, and most substitutions were synonymous; however, 10 SNPs resulted in predicted amino acid substitutions in genes associated with viral replication, transcription, membrane-associated functions, and host-interaction-related proteins.

## 4. Discussion

This case report describes the first occurrence of a GPV outbreak in captive wild ruminants, affecting Barbary sheep, Nubian ibex, Arabian oryx, and Scimitar oryx. The outbreak highlights the capacity of capripoxviruses to cross species barriers and establish clinically significant disease beyond their traditional domestic host.

The severity of the disease differed markedly between species and appeared more pronounced in adult animals compared to juveniles. Barbary sheep exhibited the highest morbidity rate, whereas Nubian ibex (genus Capra) and Arabian oryx experienced very high mortality rates. Scimitar oryx demonstrated a comparatively higher rate of survival. These interspecies differences likely reflect a combination of host susceptibility, population density, and additional behavioral patterns rather than a strain-specific virulence alone.

Recent reviews on capripoxvirus infections have highlighted that natural infections in wildlife, although rare, have been reported. In India, GPV has been documented in wild species such as the red serow and Himalayan goral [8,9]. The apparent scarcity of reports may therefore reflect a combination of factors, including limited surveillance in wildlife populations, underdiagnosis of sporadic cases, or lack of molecular confirmation, rather than true absence of susceptible host species, as several wild ruminants have been shown to be susceptible to capripoxvirus infections [10,11]. It should also be noted that one report from the region suggests that similar outbreaks in wild or semi-captive ruminants may have occurred previously but were not formally published. For example, a GPV outbreak affecting wild ruminants in Qatar was presented at a scientific conference (Sharjah International Conservation Forum for Arabia’s Biodiversity 2018) several years ago, but no peer-reviewed publication is currently available. This further supports the notion that GPV spillover into wildlife may be underrecognized rather than truly absent.

The GPV outbreak in the UAE followed a period of unusually heavy rainfall, raising questions about environmental factors that may have facilitated the viral emergence and spread. Although the reserve was fully enclosed and strict biosecurity measures were implemented for staff and vehicles, the source of the outbreak remains unclear. Indirect contact with domestic small ruminants through the perimeter fencing cannot be excluded. Small farms with free-grazing sheep and goats are common in the surrounding area, although direct interaction is considered unlikely. Environmental conditions may have contributed to viral transmission. Heavy rainfall combined with warm temperatures can enhance breeding conditions for insect vectors, such as *Culicoides* spp., *Stomoxys calcitrans,* and mosquitoes, which are known to act as mechanical transmitters of capripoxviruses [5,12]. Additionally, high animal density within the fenced reserve, particularly around the communal feeding stations during hot periods, likely facilitated direct contact transmission.

The general health as well as the nutritional status of the affected animals may also have influenced disease outcome. Even though no systematic assessment of body condition, parasitological burden, or concurrent infectious diseases was performed during the outbreak, postmortem examinations did not reveal significant parasitism or additional disease processes. Nevertheless, the unusually heavy and long rainfall preceding the outbreak may have reduced forage quality and increased energetic demands, potentially leading to transient nutritional stress or immunosuppression at the population level. Such environmental factors may have increased susceptibility to infection or exacerbated disease severity once GPV was introduced.

Whole-genome sequencing demonstrated that the isolates from Barbary sheep and Arabian oryx were genetically identical, supporting a single introduction event followed by intra-reserve transmission. Phylogenetically, the UAE isolate clustered with the Central Asia and Western Asia Line and showed close similarity to the Turkish field strain, with 60 base pairs of differentiation. This strain from Turkey was recently sequenced [13]. Unfortunately, it is unclear in which year the sequenced GPV strain was isolated. This close genetic relationship suggests regional circulation of related GPV strains rather than the emergence of a novel, highly divergent variant. The high mortality observed in some species is more likely attributable to host-specific susceptibility and ecological conditions rather than enhanced viral virulence.

The outbreak illustrates the vulnerability of enclosed wildlife populations to livestock-associated pathogens. In semi-captive systems with limited immigration and emigration, infectious disease introduction can have a rapid and significant consequence. Although temporary, a reduction in population density may reduce competition; the ecological impact, including effects on predator-prey dynamics, vegetation pressure, and genetic diversity, may be substantial in the long term. No obvious long-term effects on reproductive success or general fitness were observed in surviving animals during the limited post-outbreak observation period. It can be assumed that extreme weather events, such as prolonged heavy rainfall, will occur more frequently worldwide [14]. This may further increase the risk of infection by vectors infected with capripoxvirus in susceptible domestic and wild animals.

Laboratory confirmation of capripoxvirus infections in wild animals is diagnostically challenging. Histopathology may be inconclusive when characteristic intracytoplasmic inclusion bodies are absent, as their presence depends on the lesion stage and tissues sampled. Virus isolation proved difficult and was successful only in lamb testis cells despite high viral load in tissues. This underscores the importance of molecular diagnostics, particularly real-time PCR, as the most reliable method for confirmation. Whole-genome sequencing of isolates enhances epidemiological tracing and strain characterization in outbreaks [13,15,16].

Preventive strategies must focus on the livestock–wildlife interface. Live attenuated vaccines against GPV and SPV are widely used in endemic regions and provide effective, long-lasting immunity in domestic ruminants [3,4]. Vaccinations of surrounding sheep and goat populations could establish a buffer around wildlife reserves and reduce the risk of future spillover. In endemic regions, routine vaccination prior to increased vector activity time may be beneficial.

Vaccination of wild ruminants presents greater challenges. Capture and handling of free-ranging or semi-captive animals, especially in mountainous areas, can cause significant stress and carry risks of injuries and mortalities. Darting may be feasible only for selected or highly endangered species, but large-scale immunization is logistically challenging and cost-intensive. In addition, safety and immunogenicity data for existing live attenuated capripoxvirus vaccines in non-domestic species are limited. Therefore, risks include adverse reactions, an insufficient immune response, or the circulation of vaccine-derived virus strains in wildlife populations. For these reasons, vaccination of wildlife needs to be carefully evaluated on a case-by-case basis and should be supported by species-specific data.

Given these constraints, strengthening livestock vaccination in surrounding areas likely represents the most practical prevention method.

Several limitations need to be acknowledged. The study is based on an observational outbreak investigation without experimental controls, limiting the ability to establish causal relationships between environmental factors, host condition, and disease severity. A serological survey of surviving animals was not performed, and pre-existing immunity within the population could not be assessed. In addition, no vector surveillance or pathogen detection in insects was conducted, and environmental samples were not analyzed. leaving transmission pathways largely inferential. No samples were obtained from nearby domestic livestock, preventing confirmation of the suspected spillover origin. Furthermore, not all deceased animals underwent full necropsy, and histopathological evaluation and detailed laboratory analyses were limited to a small number of individuals, which may not fully capture interspecies variability in disease progression. The absence of longitudinal follow-up also precludes the assessment of long-term outcomes or immunity in the surviving animals. Finally, as the outbreak occurred within a single fenced reserve, the generalizability of these findings to other wildlife systems may be limited. Despite the limitations, clinical presentation, high viral loads in real-time PCR, and whole genome sequencing collectively provide strong evidence that GPV was the cause of the outbreak.

## 5. Conclusions

This case series documents the first confirmed outbreak of GPV in captive wild ruminant species, including Barbary sheep, Nubian ibex, Arabian oryx, and Scimitar oryx. The findings highlight the potential for capripoxviruses to cross species barriers and establish infection beyond traditional domestic hosts. It also underscores the importance of integrating wildlife health monitoring into regional livestock disease control strategies. Capripoxviruses remain endemic in parts of the Middle East and Central Asia, and their capacity to affect wildlife populations adds a new dimension to disease management.

Effective prevention of future outbreaks requires coordinated surveillance, rapid molecular diagnostics, comprehensive livestock vaccination strategies, and strong cross-sector collaboration among veterinary authorities and wildlife management to protect biodiversity and agricultural sustainability.

## Figures and Tables

**Table 1:** Number of animals affected listed by species. Morbidity and case fatality during the goatpox virus outbreak in the period from 20 May 2024, to 10 August 2024.

No	Species	Total Number of Animals in Reserve	Total Number of Animals Clinically Affected Animals	Case Fatality (Died/Euthanized)	Adult	Young	Morbidity Rate *	Case Fatality Rate Among Affected Animals **
1	Barbary sheep (*Ammotragus lervia*)	1100	495	54	51	3	45%	11%
2	Nubian ibex (*Capra nubiana*)	215	8	8	8	0	4%	100%
3	Arabian oryx (*Oryx leucoryx*)	35	6	6	6	0	20%	100%
4	Scimitar oryx (*Oryx dammah*)	62	7	3	3	0	13%	43%
Total			71	71	68	3		

* Morbidity Rate: clinically affected animals/total animals in the reserve x 100. ** Case Fatality Rate: animals that died or were euthanized/clinically affected animals x 100.

**Table 2:** Real-time PCR results of original tissue samples.

		Pan-Capripox-p32	Beta-Actin	LSDV-Field	LSDV-VAC	SPV	GPV
Host	Specimen	FAM	HEX	FAM	HEX	FAM	HEX
Barbary sheep	skin	22.8	31.9	N/A	N/A	N/A	24.0
	lung	28.2	29.2	N/A	N/A	N/A	30.5
	Ln. prescapularis	27.1	30.2	N/A	N/A	N/A	28.5
Arabian oryx	skin	18.4	N/A *	N/A	N/A	N/A	20.1
	lung	16.1	N/A *	N/A	N/A	N/A	17.4
	Ln. prescapularis	23.3	27.1	N/A	N/A	N/A	25.4

Semi-quantitative evaluation of Ct values: >40 = N/A = negative; 35 to 40 = questionable/borderline; 30 to 35 = weakly positive; 25 to 30 = moderately positive; 20 to 25 = strongly positive; <20 = very strongly positive. * negative results of internal process control due to competitive inhibition by very strong positive results in the Pan-Capripox assay.

## Data Availability

The original contributions presented in this study are included in the article. Further inquiries can be directed to the corresponding authors.
